# Modelling  vaccination schedules for a cancer immunoprevention vaccine

**DOI:** 10.1186/1745-7580-1-5

**Published:** 2005-10-07

**Authors:** Santo Motta, Filippo Castiglione, Pierluigi Lollini, Francesco Pappalardo

**Affiliations:** 1 Department of Mathematics and Computer Science, University of Catania, Catania, Italy; 2 Sezione di Cancerologia, Dipartimento di Patologia Sperimentale, University of Bologna, and Centro Interdipartimentale di Ricerche sul Cancro "Giorgio Prodi", Italy; 3 Istituto per le Applicazioni del Calcolo, Consiglio Nazionale delle Ricerche, Roma, Italy; 4 Faculty of Pharmacy, University of Catania, Italy

## Abstract

We present a systematic approach to search for an effective vaccination schedule using mathematical computerized models. Our study is based on our previous model that simulates the cancer vs immune system competition activated by tumor vaccine. This model accurately reproduces *in-vivo *experiments results on HER-2/neu mice treated with the immuno-prevention cancer vaccine (Triplex) for mammary carcinoma. In vivo experiments have shown the effectiveness of Triplex vaccine in protection of mice from mammary carcinoma. The full protection was conferred using chronic (prophylactic) vaccination protocol while therapeutic vaccination was less effcient.

In the present paper we use the computer simulations to systematically search for a vaccination schedule which prevents solid tumor formation. The strategy we used for defining a successful vaccination schedule is to control the number of cancer cells with vaccination cycles. We found that, applying the vaccination scheme used in *in-vivo *experiments, the number of vaccine injections can be reduced roughly by 30%.

## 1 Introduction

Tumor immunologists have long sought ways to turn experimental results into effective therapies for human cancer patients. The best results so far are provided by using various monoclonal antibodies directed against tumor cells, which won approval from regulatory agencies and entered clinical practice. Other approaches, such as therapeutic vaccines that aim at stimulating the immune response of the host against tumor cells, were much less successful [1]. Experimental evidence clearly shows that vaccines elicit effective responses against early, microscopic tumors, but are ineffective against established, large tumor masses. A similar situation is found in infectious immunity: prophylactic vaccines protect millions of individuals worldwide from pathogens, whereas therapeutic vaccines are mostly ineffective. Such results led some tumor immunologists to the idea that the effort should be directed towards the development of prophylactic, rather than therapeutic, cancer vaccines [2]. Prophylactic vaccines against viruses that increase the risk of cancer, such as hepatitis B virus (HBV) or papilloma virus (HPV), have already shown a significant effcacy in reducing cancer incidence [3,4]. The challenge is now to devise immunological strategies for the design of prophylactic vaccines for prevention of those human cancers that are not related to viral infections.

Standard vaccines against viruses induce the primary response of the adaptive components of the immune system against the non-self antigen in order to activate the immune system memory which will then elicit the much stronger secondary response when the antigen will enter the body. There is no need to break self tolerance as the antigen is non self.

At variance tumor vaccines need to break tolerance of the tumor associated antigens (TAA) that otherwise would be treated as self and not attacked by the immune system.

The effectiveness of tumor immuno-prevention was demonstrated in the past ten years in several mouse models of tumor development. Tumors in these models are induced either by chemical carcinogens or by transgenic expression of oncogenes. Among the most thoroughly investigated is the HER-2/neu transgenic mouse model. HER-2/neu is an oncogene involved in human breast and ovary carcinomas. The protein product of HER-2/neu, p185neu, is a membrane tyrosine kinase that transduces proliferative signals. A deregulation of HER-2/neu, for example due to gene amplification, leads to uncontrolled cell proliferation. Over-expression of a rat HER-2/neu transgene in mice was obtained using tissue-specific regulatory sequences derived from mammary tumor virus (MMTV) long terminal repeats (LTR). Various transgenic mouse lines were obtained using either a mutant, constitutively activated HER-2/neu gene or a normal, non mutated gene. Here we will refer to mice carrying the mutant oncogene, which are prone to a very aggressive mammary carcinogenesis, invariably leading to the development of invasive mammary carcinomas by the age of 6 months. The natural history of mammary carcinoma in HER-2/neu transgenic mice was thoroughly investigated and found to be remarkably similar to that of human breast cancer [5]. Immuno-prevention of mammary carcinoma in HER-2/neu transgenic mice was attempted using various immunological strategies, including cytokines, non-specific stimulators of the immune response, and HER-2/neu specific vaccines made of DNA, proteins, peptides, or whole cells. Most approaches achieved a delay of mammary carcinogenesis, but a complete prevention of tumor onset was not attained, particularly in the most aggressive tumor models [6]. We achieved the first complete success at preventing mammary carcinoma in HER-2/neu transgenic mice using a vaccine that combined three different stimuli for the immune system. The first was p185neu, protein product of HER-2/neu, which in this system is at same time the oncogene driving carcinogenesis and the target antigen. p185neu was combined with the two non specific adjuvants, allogeneic class I major histocompatibility complex (MHCI) glycoproteins and interleukin 12 (IL-12). MHCI glycoproteins are responsible for some of the most intense immune responses observed during the rejection of allogeneic organ transplants. Unlike conventional antigens, allogeneic MHCI molecules stimulate a relatively large fraction of all T cell clones, up to 10% of the available repertoire. IL-12 is a cytokine normally produced by antigen presenting cells (APC) such as dendritic cells (DC) to stimulate T helper cells and other cells of the immune system, such as natural killer cells (NK) [7]. IL-12 was initially administered systemically, but a more recent formulation of the Triplex vaccine used genetically modified vaccine cells transduced with IL-12 genes, thus allowing cytokine production at locally high levels that more closely mimicked the natural release of IL-12 [8]. The vaccine cell (VC) that we will model here is similar to the latter one and consists of a HER-2/neu transgenic mammary carcinoma cell allogeneic with respect to the host and transduced with IL-12 genes.

**Figure 1:**
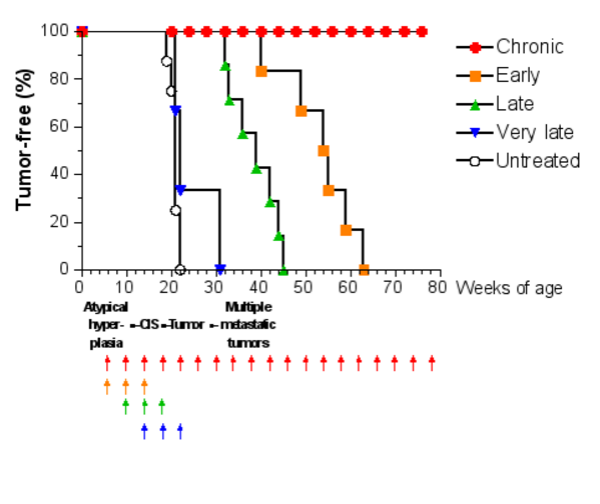
Tumor-free survival curves of HER-2/neu transgenic mice receiving the Triplex vaccine according to different protocols from [6]. Each arrow at the bottom of the graph represents one cycle of vaccination. The sequence of neoplastic progression in untreated mice is outlined under the x axis; CIS, carcinoma in situ.

A complete prevention of mammary carcinogenesis with the Triplex vaccine was obtained when vaccination cycles started at 6 weeks of age and continued for the entire duration of the experiment, at least one year (chronic vaccination). One vaccination cycle consisted of four intraperitoneal administrations of non-replicating (mitomycin-treated) VC over two weeks followed by two weeks of rest [8]. We made various attempts at reducing the number of vaccination cycles, in particular we studied the effects of just three cycles starting at 6 weeks of age (early vaccination), or at 10 weeks of age (late vaccination), or at 16 weeks of age (very late vaccination). Early vaccination produced a significant delay in the onset of tumors, but all mice eventually succumbed to mammary carcinoma. Late vaccination was less effective than early vaccination, whereas the very late protocol was completely devoid of effect in comparison to untreated control mice (Figure 1).

The question whether the Chronic protocol is the minimal vaccination protocol yielding complete protection from tumor onset, or whether a lower number of vaccination cycles would provide a similar degree of protection is still an open question. Finding an answer to this question via a biological solution would be too expensive in time and money as it would require an enormous number of experiments, each lasting at least one year. For this reason we developed an accurate model of immune system responses to vaccination and we use this model as a *virtual laboratory *to search for effective vaccination protocols. The paper is organized as follows: section §2 will introduce the general requirements needed to model the problem; section §3 describes in details our *virtual laboratory*, i.e. the model, its structure and biological details, and its computer implementation (the simulator). In section §4 we present *in silico *experiments for different vaccination schedules and we show how the *virtual laboratory *can be used for designing a new and better protocol. Finally in section §5 we discuss our results, draw some conclusions, and plans for future investigations.

## 2 The general framework for modelling

In dealing with modelling of cancer – immune system interaction and competition one should be aware that this competition can play a crucial role besides therapeutical actions. This competition may possibly end up either with the elimination of the cancer cells, or the progressive invasion by cancer cells of other tissues or organs.

The goal of medical treatments is to enhance the immune response by activating the immune defense and/or specializing the ability of immune cells to identify the presence of the tumor.

The immune competition is a phenomenon which involves aggressive cells or particles (either external non-self pathogens or self modified or corrupted cells) and the various populations of the immune system. The immune system appears to be a distributed system which lacks central control, but which, nevertheless, performs its complex task in an extremely effective and effcient way. Complexity, in this framework, is driven by the fact that interactions are developed at different scales (i.e. the cellular dynamics is ruled by sub-cellular interactions) and different mechanisms operate on the same subject (mechanical for the dynamics and biological for the immune competition). The state of the art of the immune mechanisms from the view point of molecular biology are described in specialized literature [9-11]. Owing to the rapid progress of biological knowledge this is rapidly changing.

A model, which is a mapping from a real-world domain to a mathematical domain, highlights some of the essential properties while ignoring not relevant (or believed not relevant) ones. A good model must be **relevant**, capturing the essential properties of the phenomenon, **computable**, driving computational knowledge into mathematical representation; **understandable**, offering a conceptual framework for thinking about the scientific domain; and **extensible**, allowing the discovery of additional real properties in the same mathematical framework.

In the framework of immune system competition, relevant means that a model should be able to capture the essential properties of the system, the system entities organization and their dynamic behavior. A computable model should then be able to simulate both the dynamic behavior and the evolution and interactions of the system entities which play the game. An understandable model must reproduce concept and ideas of tumor immunology while opening new computational possibilities for understanding the immune competition. Finally extensible models should allow the inclusion of new knowledge with a limited effort. The immune system is characterized by a great complexity so that it is very diffcult, or even impossible, to develop a detailed mathematical description of all phenomena related to the immune competition which satisfies all the above properties. A significant effort has recently been devoted in searching for an appropriate mathematical approach to describe the Immune System – tumor competition (see [12] for a recent review). However, if one focuses the attention on specific types of interactions, one may attempt to develop *ad hoc *models for a specific phenomenon at the chosen observation and representation scale. Extensive description of subcellular vs larger scales of modelling can be found in [13-15]. Methods based on generalized kinetic model look very promising for system description: however they are not yet so detailed to model a specific therapeutic vaccine.

## 3 SimTriplex Model

To model the action of a specific tumor vaccine, we used a computational approach which reproduces the *ab initio *kinetic model that describes the interactions and diffusion of each relevant biological entity. This approach is biologically very flexible; the behavior of entities is modelled using present biological knowledge and can be easily modified to reflect observations from new biological experimental results. Compared to the complexity of the real biological system our model is still very *naive *and it can be extended in many aspects. However the model is complete enough to describe the major aspect of the phenomenon and, after tuning the model parameters, it can predict the response to a vaccination schedule that prevented the formation of solid tumors in mice.

Our model, hereafter referred as SimTriplex model, describes the immune competition using an agent based method. These methods are nowadays very popular as they find application in various fields. However the idea of discrete agents whose global dynamics is able to reproduce macroscopic behavior has been introduced more than fifty years ago in the framework of fluid-dynamics.

We used a Lattice Gas Automata (LGA) approach which allows to describe, in a defined space, the immune system entities with their different biological states and the interactions between different entities. The immune system evolution in a 2D physical space and in time is generated from the interactions and diffusion of the different entities. Extension to a 3D physical space is possible, but it would obviously have an higher computational cost. The major advantage of this technique is that the entities and the relationships can be described in terms that are much similar to the biological world. The intrinsic non linearity of the system is treated with no additional efforts. Models based on this class of techniques reproduce the biological knowledge of the system; so they are relevant, understandable and extensible. They are naturally computable but the computational effort increases drastically with increasing biological details (e.g. the immune repertoire and biological details). This class of model can be seen as the computational counterpart of the generalized kinetic model.

To describe the cancer – immune system competition one needs to include all the entities (cells, molecules, adjuvants, etc.) which biologists recognize as relevant in the competition. These entities have internal states, birth, age and death (i.e. ages structure). Interactions between different entities will stochastically change the internal state of one or both the interacting entities. Space changes in the system are achieved with diffusion instead of collision (see 3.2.4). It is worth to remind that in the microscopic cellular framework one is interested only in the initial and final state. The model of the mechanisms which produce the change of state is deferred to sub-cellular models.

Our model is driven by the experimental data on Triplex, an engineered vaccine for mammary carcinoma tested on HER-2/neu transgenic mice.

The model, which has been fully described in [16] include the entities described in table 1. Here we want to recall entities representation in order to highlight the computational approach.

**Table 1:** List of symbols and half life. Half life is expressed in times teps; one times tep represents 8 hours.

entity	symbol	half life
B Cells	B	3.33 days
Antibody Secreting Plasma Cells	PLB	10
T-helper lymphocytes	TH	10
T-cytotoxic lymphocytes	TC	10
Macrophages	MP	3.33 days
Dendritic Cells	DC	10
Interleukin-2	IL-2	5
Immunoglobulins	IgG	5
Danger Signal	D	5
Major Histocompatibility Complex Class I	MHCI	-
Major Histocompatibility Complex Class II	MHCII	-
Tumor Associated Antigens	Ag	3
Immunocomplexes	IC	100

Cancer Cells	CC	1095
Vaccine Cells	VC	5
Natural Killer Cells	NK	10
Interleukin-12	IL-12	5

### 3.1 Entities Representation

The primary function of the immune system is a *recognition *function. Working at cellular scale we need to represent only those characteristics of entities which are relevant for recognition and interaction: state, recognition site and lifetime.

From the computational point of view, the major difference between cellular and molecular entities is that cells have a state attribute and may be classified on the basis of these attributes. Entities with no biological state, like antibodies are not followed individually, instead we consider only their total number for each lattice point. Their age structure is obtained increasing or decreasing the population number. Antigens (Ag or TAA) are however followed as individuals for their specific role.

Entities with biological states, i.e. cellular entities, are tracked individually in order to keep track of state change owing to interactions. The state of a cell is an artificial label introduced by the logical representation of the cells behavior. They have a standard lifetime which can eventually increase with interactions.

Most cellular and molecular entities have recognition sites (receptors). The set of lymphocyte's receptors is represented by bit-strings of length *h *[17] which then forms the so called "shape space" [18]. A clonal set of cells is characterized by the same clonotypic receptor, i.e. by the same bit-string of length *l*. The potential repertoire of receptors scales as 2^*l*^.

Simple entities, like antibodies, are represented using their binding site (receptors). Tumor associated antigens are also described using their binding site but we keep also track of their lifetime. Representation of cellular entities include receptors, binding site and lifetime.

The receptor-coreceptor binding among the entities are described in terms of **matching **between binary strings with fixed directional reading frame. Bit-strings represent the generic "binding site" between cells (through their receptors) and target molecules (through peptides and epitopes). Every cellular entity is represented by a number of molecules, including the receptor. The repertoire is then defined as the cardinality of the set of possible instances of entities that differ in, at least, one bit of the whole set of binary strings used to represent its attributes.

Indeed, the cells equipped with binding sites and the antibodies, have a potential repertoire of , where *N*_*e *_indicates the number of binary strings used to represent receptors, MHC-peptide complexes, and epitopes of the entity *e*. Other entities do not need to be specified by binary strings so their repertoire is represented by a single entity (i.e. *N*_*e *_= 0). Examples include cytokine molecules such as interferon-*γ *and the danger signal [19].

**Figure 2:**
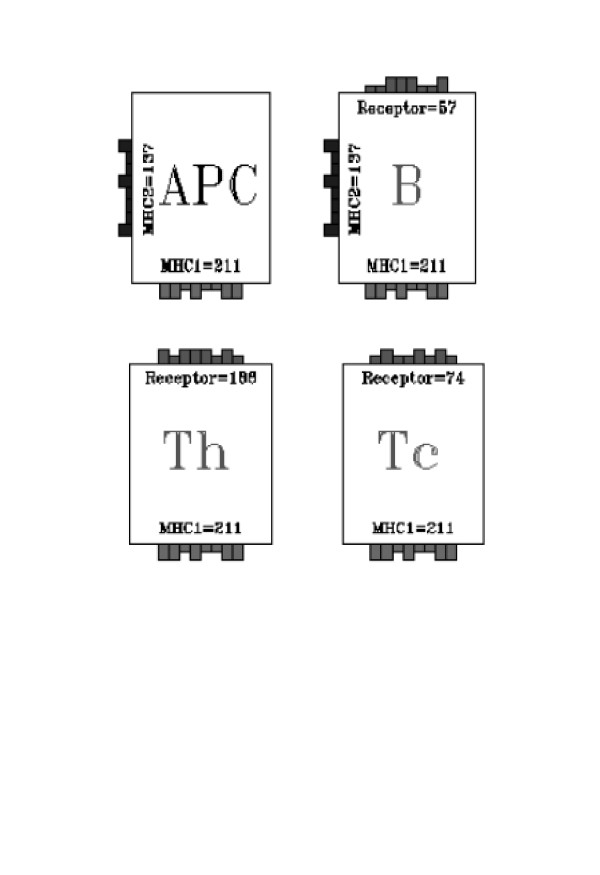
Grafical representation of lymphocytes and antigen presenting cells.

**Figure 3:**
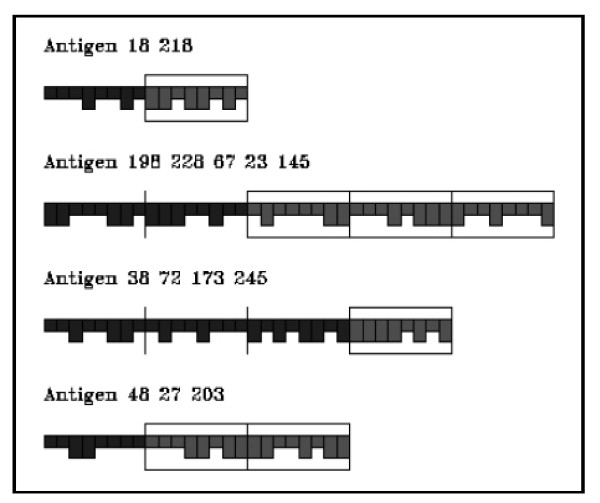
Graphical representation of antigens.

Figures 2 and 3 show a graphical representation of lymphocytes and antigens. The bit-string is labelled with its decimal representation. The figures are taken from ImmSim v.3 userguide ^1^.

As receptors' entities are represented by bit strings, the only information available is a "similarity" between bit strings. A standard measure of similarity between two bit strings is the so called Hamming distance which is just the number of mismatching bits ^2^. The interactions between two entities equipped with receptors are defined by a probability measure, called **affnity potential**, which is a function of the **Hamming distance **between the binary strings representing the two entities' binding site. For two strings *s *and *s*' such probability is max when all corresponding bits are complementary (0 ↔ 1), that is, when the Hamming distance between *s *and *s*' is equal to the bit string length. If *l *is the bit string length and *m *is the Hamming distance between the two strings, the affnity potential is defined in the range 0, . . ., *l *as



where *v*_*c *_∊ (0,1) is a free parameter which determines the slope of the function where as *m*_*c *_(*l*/2 <*m*_*c *_≤ *l*) is a cut-off (or threshold) value below which no binding is allowed.

The cells are free to diffuse across the lattice sites. At each time-step, representing 8 hours of real time, the cells and molecules residing on the same lattice site they can interact each other. The tissue is represented as two-dimensional triangular lattice (six neighbor sites) *L *× *L*, with periodic boundary conditions in both directions (up-down, left-right). The lattice is taken to represent here a portion of mammary tissue of the mouse.

All various classes of immune functional activity, phagocytosis, immune activation, opsonization, infection and cytotoxicity are described by computational rules.

The model also includes the mechanism of haematopoiesis as described in [16]. The rules which implement these actions are executed in a randomized order.

### 3.2 Coding SimTriplex

The high-level architecture of the code can be explained using the following pseudo-code.

   **SimTriplex Simulator**

   **Input: **Accept pre-determined inputs (e.g., user-specified vaccine injections, random generation of new B- and T-cells, etc.).

   **Time cycle start**

   **Activate Thymus and Bone Marrow functions.**

   Haematopoiesis function is activated and operates for all cycles.

   **Interaction-driven dynamics. **Enables the pairing of entities and modification of their states, creates new entities in response to these interactions, etc. in response to these interactions.

   **Internal dynamics. **Makes allowance for internal, non interaction-driven dynamics (e.g. aging and natural death, differentiation, mutation, etc.).

   **Diffusion. **Allows cellular entities to diffuse on the lattice checking the physical space constraints. Change the density of molecular entities in order to mimic diffusion.

   **Output. **Stores a trace of the state of the system at time *t*.

   **Time cycle ends**

   **code end**

A set-up routine will create the lattice, register the values of the various input parameters and stochastically fill the lattice with all population of cells. Time step interactions will proceeds up to a preselected final time.

Cellular and molecular entities are treated differently in the model. Cellular entities are treated individually while molecular entities are modelled as populations. We describe them separately.

#### 3.2.1 Cellular entities

The common features of cellular entities are : Position, Specificity, States and Age.

Position is the crucial parameter which defines which entities can interact as interactions between entities can occur only between those entities which are co-located in the same lattice cell;

In the *shape space *[17] entities interact according to their specificity represented by binary string. This simulates recognition of the entities by their paratopes (complementary shapes of peptides and receptors). Each entity, except for plasma cells, has at least one receptor (or paratope/epitope, depending on whether they are cellular or molecular entities) which is its primary identifier, i.e., its specificity.

The key constituents of specificity are MHC class I (MHC-I) and MHC class II (MHC-II), T cell receptors (TCR) and B-cell receptors (BCR). The different class of cellular entities we consider are: B-cells, T helper cells (TH), cytotoxic T cells (TC), antigen presenting cells (APC) and plasma cells (PLB). B cells are endowed with MHC-II and BCR; TC cells are equipped only with TCR (CD8) and TH include TCR (CD4).

With the name APC cells one refers to Macrophages (MP), Dendritic cells (DC) and B-cells. MP are aspecific APC and do not contain any specific receptor. They phagocytose antigens (in our case TAA) and expose their bit string as MHC-II. DC are also aspecific APC. They internalize TAA and expose as MHC-I and MHC-II. Finally B-cells act as specific APC. If they recognize TAA they internalize it and expose as MHC-II. Plasma cells have no specific receptor (but contains the information of the original BCR). They produce antibodies with the same receptor as the B cell from which they originate.

Internal states of each cell type are summarized in Table 2.

**Table 2:** Entities' states. Internal states (columns) of each cell type (rows) are labelled with a •. The rows show the states of each entity.

Cell	Active	Re-sting	Intern	PresI	PresII	Duplica	Bound-ToAb
B	•	•	•		•	•	
TH	•	•				•	
TC	•	•				•	
DC	•	•	•	•	•		
MP	•	•	•		•		
CC				•		•	•
VC				•			•
NK	•						

Each cells can be in different internal states and all cells are tracked individually throughout the course of an experimental run. In order to describe state changes we will look (see § 3.2.3) at the evolutions of each cellular entity since its initially entry in the lattice. Here we briefly describe the biological meaning of entities states.

APC, TH and B cells are initialized as **Active**; TC cells are initialized as **Resting**; VC are initialized as **PresI **while CC are stochastically set as **PresI **with an high probability. All entities are initialized with their default life time (see **Age**).

All cellular entities will then change their status following a successful interaction with another entity (hereafter referred as positive interaction) or by internal processing. Conditions under which a positive interaction (or the opposite negative interaction) occurs will be described later (see § 3.2.3).

• APC (MP, DC and B cells) will change their status to **Intern **when they interact positively with TAA; Internal processes will then change the state to **PresI **(MHC class I) or **PresII **(MHC class II). DC may present both in MHC-I and MHC-II. Positive interaction of a presenting MHC-II APC with an active TH cell will change both B cells APC and TH state to **Duplica **and clonal proliferation phase begins for B cells. If a **PresII **APC negatively interacts with a TH cell, its status can return to **Active**. This simulates the biological event that a presenting APC that is not stimulated by TH-cell may loose the presentation status.

• B cells change their status only when they positively interact with a TAA or with a TH cell as described above.

• Cytotoxic T cells become **Active **when positively interact with a presenting MHC-I DC, or with a VC in presence of IL-12 adjuvant or with a CC in presence of IL-2 previously released by an activated TH cell. Active TC cells can recognize and kill a CC by lysis. In such a case, the status of the TC changes to **Duplica **and clonal division starts.

• Helper T cells change their status only when they positively encounter an APC as described above.

• Cancer and vaccine cells positively interact with Antibodies and change their status to **BoundToAb**.

Antibodies can then kill them by complement mediated cytotoxicity or can act as signals for NK cells. Cellular entities have age structure. They are born, they interact and duplicate (and eventually get anergic) and, after a finite lifetime, they die by apoptosis or by lysis. We need to keep track of the age of cellular entities; we do this by keeping a count of the number of time-steps since cell birth (from stem cells or by clonal division). To simulate memory cells, we increase the halflife of TH, TC and B cells after successful interaction with target antigens. The death probability reaches 1 when the age gets to twice the half life. The simulator performs the process of the thymic selection of T cells (TH + TC) as described in [16]. Selection has two phases: negative and positive (both stochastic).

The simulated immune system achieves repertoire completeness by a mechanism of mutation of B-cell receptor (BCR). Mutation occurs when a new B cell is formed, i.e. in the duplication phase. This effect is included in the simulator as a stochastic event; this increases the immune system recognition ability. The setup routine initially set entities concentration in the lattice. The Leukocytes form three general classes (Granulocytes; Lymphocytes and Monocytes) which are present in different ratios in blood, tissues and various organs. In our virtual mouse we initially consider 4500 leukocytes. Of these there are 4200 lymphocytes, comprising 1512 B cells, 1512 T cells (subdivided in 1008 T helper cells and 504 cytotoxic T cells) and 1176 natural killer cells. There are 300 monocytes divided in 150 macrophages and 150 dendritic cells. The model does not have any granulocyte.

#### 3.2.2 Molecular entities

Molecular entities included in the simulator are Antigens, Antibodies, Cytokines and Damage (Danger Signal, see [19]). Molecular entities do not have internal states and thus do not need to be modelled individually (only TAA are treated individually). Rather, we define populations which represent different specificity of molecular entities, on the lattice. Diffusion on the lattice is performed by appropriate change of the concentrations of entities on the lattice. The age structure is stochastically increased/decreased as function of production and half life time respectively.

As antigen we consider only the tumor associated antigen (p185) released by injected vaccine cells and cancer cells when they die. Antigens are represented by a number of binary segments consisting of a fixed number of bits. These segments represent the antigenic sites (epitopes and peptides) as shown in Figure 3. Epitopes are defined as the external portion of an antigen that is recognized by a B-cell receptor. Peptides are defined as portions of an antigen that can be bound by an MHC molecule and be recognized by an appropriate T cell. The epitopes and peptides are specified separately in the model. The minimum effective antigen we consider is therefore two segments long, one for B-cell epitope and one for peptide.

Antibodies (Ab) are also represented by bit strings, and have a paratope which is identical to the paratope/receptor of the B cell (plasma cell) that secretes them.

Cytokines (IL-2 and IL-12) and a danger signal are encoded as aspecific entities. Their presence in the lattice site will induce or prevent interactions by increasing or decreasing probabilities.

#### 3.2.3 Interactions

In this section we will describe interactions following the evolution of the simulator for the first few steps. First of all we must remember that interactions occurs only if two entities stay in the same site. Taking into account that a time step is 8 hours we can say that entities in a site are those entities that a single entity encounters during 8 hours. Time t = 0 corresponds to the atypical hyperplasia, i.e. first appearing of tumor cells. For "early schedule" time t = 0 corresponds also to the first vaccine injection. An interaction between two entities is a complex action which eventually end with a state change of one or both entities. Interactions can be *specific *or *aspecific*. Specific interactions need a *recognition phase *between the two entities (e.g. B ↓ TAA); recognition is based on Hamming distance and affnity function and is eventually enhanced by adjuvants. We refers to *positive interaction *when this first phase occurs successfully. Aspecific interaction do not have a recognition phase (e.g. DC ↓ TAA). When two entities, which may interact, lie in the same lattice site then they interact with a probabilistic law. All entities which may interact and are in the same site have a positive interaction.

First positive interaction is between vaccine cells and cytotoxic T cells (**TC – VC Interaction**). vaccine cells are engineered in such a way that are *presenting *MHC class I (status **Presl**) and TC are in the state in which are released from thymus (status **Active**). Then, if TC (CD8) cell receptor matches with a non-zero affnity with the allogeneic MHCI, VC dies by lysis and release TAA. Allogeneic-MHC present in the vaccine guarantees a non-zero affnity. Positive interaction produces TC duplication (state change into **Duplica**) and increase TC lifetime of one time cycle (8 hours). Once TAA are released they can interact with Antigen Presenting Cells (APC), (i.e. Macrophages (MP), Dendritic cells (DC) and B cells) or antibodies. Positive APC ↓ TAA interaction will have the following effect: *i) *TAA is ingested by APC; *ii) *APC will change state becoming presenting. A presenting APC is able to stimulate other cells (TC, TH). Stimulated TH produce Interleukin-2 (IL-2). A positive interaction TH B will change the state of the B cell into Plasma Cell (PLB) and the humoral response begin by antibodies production. This briefly describes the major interactions included in SimTriplex. They can be divided into standard interaction of the immune system (B ↓ Ag; Ab ↓ Ag; TH ↓ B; TH ↓ MP; MP ↓ IC; MP ↓ Ag; DC ↓ Ag; TH ↓ DC;) and interactions which occurs in presence of Tumor and vaccine (TC ↓ CC; Ab ↓ CC; NK ↓ CC; Ab ↓ VC; NK ↓ VC). Allowed interactions are shown in table 3.

**Table 3:** Simulator's interactions. An interaction between two entities occurs if the row/column interaction is marked with a •. The table is obviously symmetric.

Entity 1 Entity 2	B	Ag	Ab	TH	TC	MP	DC	IC	VC	CC	NK
B		•		•							
Ag	•		•			•	•				
Ab		•							•	•	•
TH	•					•	•				
TC									•	•	
MP		•		•				•			
DC		•		•							
IC						•					
VC			•		•						•
CC			•		•						•
NK			•						•	•	•

#### 3.2.4 Diffusion

All entities are allowed to move with uniform probability between neighboring lattices in the grid with equal diffusion coeffcient. In the present release of the simulator chemotaxis is not implemented.

## 4 Results

The systematic search for a new schedule is driven by the known *in vivo *results that we have reproduced with our model. In the following we first describe how we set up our *virtual lab in-silico *experiments which reproduced the in vivo results (section § 4.1); we then analyze results of the different schedules with to analyze the immune system reaction to vaccine injections (section § 4.2). Finally, by taking into account what we learned from computer experiments, we systematically search for a new schedule alternative to the chronic one, which could prevent the solid tumor formation (section 4.3).

### 4.1 Setting in silico experiments

*In vivo *experiments on sets of HER/2-neu mice have been carried, for all the schedules, up to the time in which a solid tumor is formed in the mouse. In fact, one observes that the effectiveness of vaccine action is depleted after a solid tumor is formed. In order to mimic these experiments *in silico *one needs to define a solid tumor on a lattice. Taking into account the number of simulated Immune System entities in the lattice we assume that a solid tumor is formed when the number of cancer cells in the lattice becomes greater than 10^5^.

The vaccination protocols that have been used to test the effectiveness of Triplex are described in section 1. In *in silico *experiments we try to reproduce *in vivo *experiments. For this we considered all the different protocols mentioned above and the case of untreated mice. The last one is analyzed in order to tune with experimental tumor growth and ensure that simulation shows no significant immune response.

We performed *in silico *experiments using the standard *good practice *statistical procedure: *i) *We considered a large population of individual mice. Each individual mouse is characterized by a sequence of uniform numbers which will determine the probabilistic events. *ii) *We randomly extract from this population two statistical samples of 100 individual mice (hereafter referred as *S*_1 _and *S*_2_) to perform numerical experiments.

**Figure 4:**
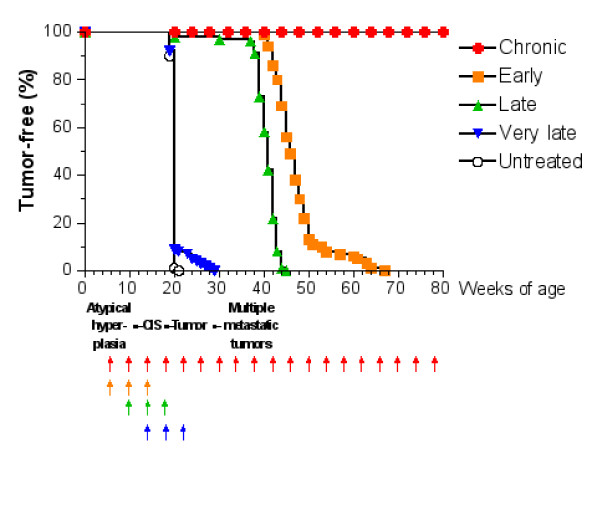
Tumor-free survival curves of virtual mice receiving the Triplex vaccine according to different protocols. Each arrow at the bottom of the graph represents one cycle of vaccination. The sequence of neoplastic progression in untreated mice is outlined under the x axis; CIS, carcinoma in situ.

The computational time begins when the mouse is six weeks old (the observed time of atypical hyperplasia) and proceeds up to the formation of a solid tumor or up to 2 years. For each protocol we treat all mice in the sample and we measure the time in which the solid tumor is formed. The percentage of tumor free mice as function of age is shown in Figure 4 for sample *S*_2 _(the same result for sample *S*_1 _has been shown in [16]). Comparison with Figure 1 shows excellent agreement with in vivo experiments.

### 4.2 Analysis of schedules' results

**Figure 5:**
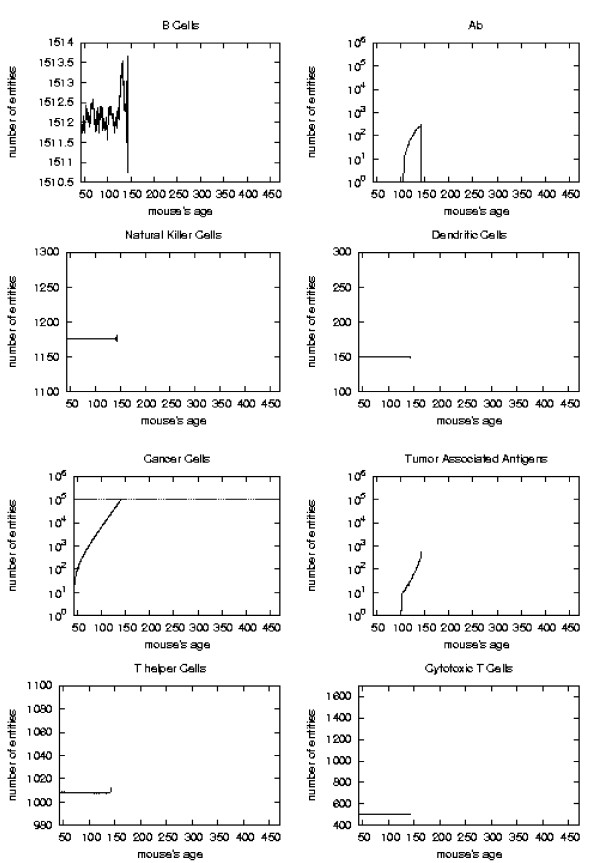
Immune response. The immune response activation is shown for untreated virtual mice, versus time in days.

**Figure 6:**
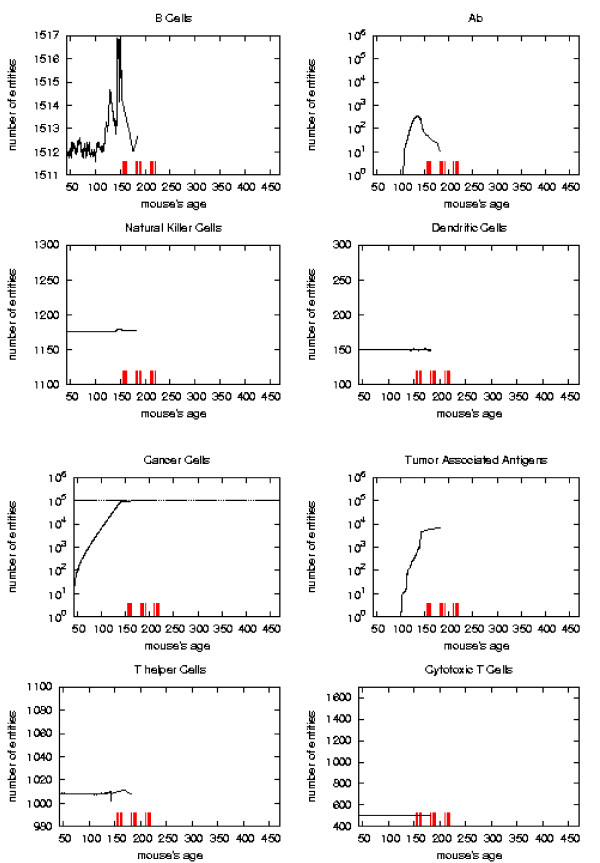
Immune response. The immune response activation due to the vaccine effect is shown for VERY LATE vaccination schedules, versus time in days. Red ticks above *x *axis represent the timing of vaccine administration.

**Figure 7:**
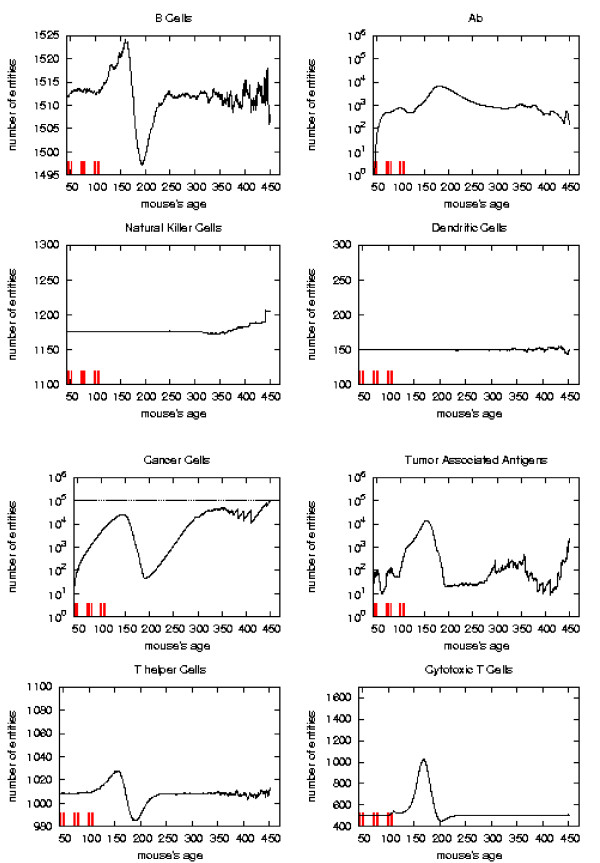
Immune response. The immune response activation due to the vaccine effect is shown for EARLY vaccination schedules, versus time in days. Red ticks above *x *axis represent the timing of vaccine administration.

**Figure 8:**
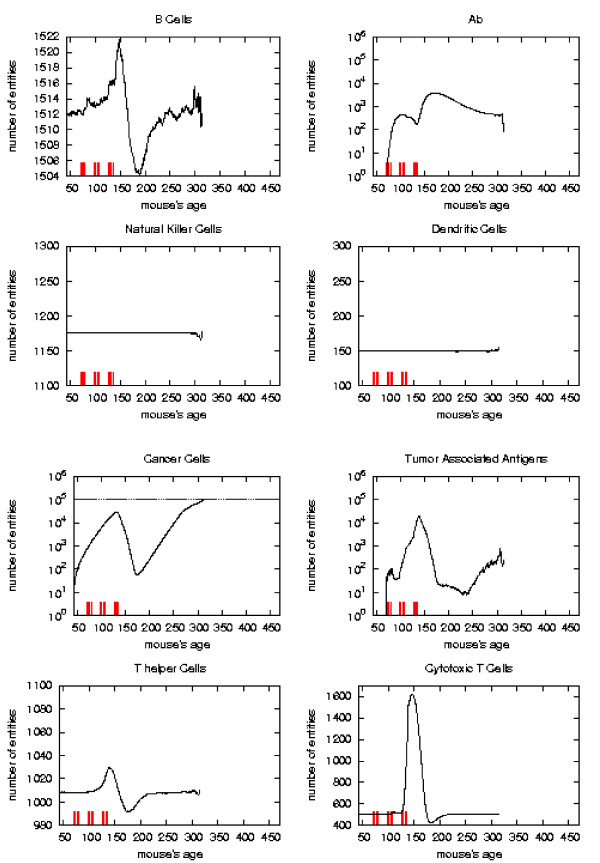
Immune response. The immune response activation due to the vaccine effect is shown for LATE vaccination schedules, versus time in days. Red ticks above *x *axis represent the timing of vaccine administration.

**Figure 9:**
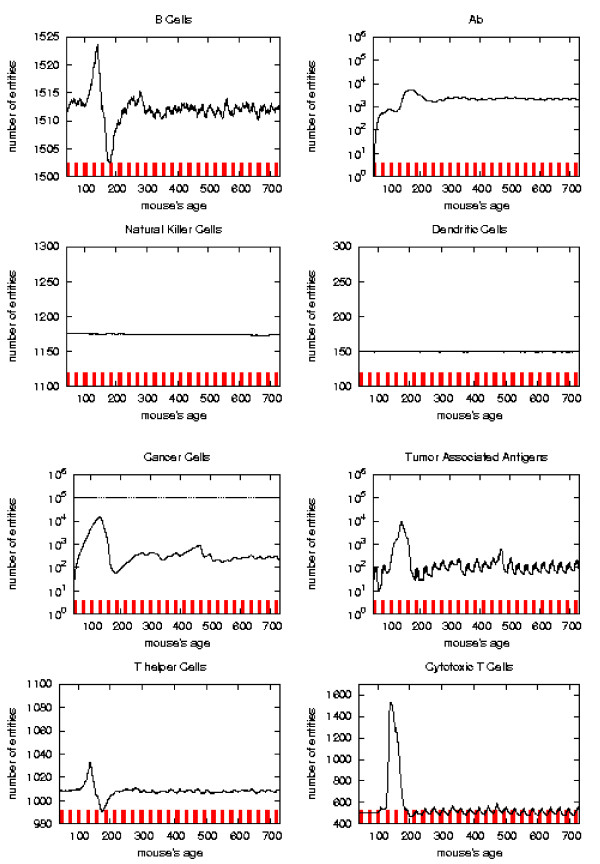
Immune response. The immune response activation due to the vaccine effect is shown for CHRONIC vaccination schedules, versus time in days. Red ticks above *x *axis represent the timing of vaccine administration.

The general behavior of the most relevant quantities versus time has been already described in [16]. Here we take the matter again to analyze in detail the system reaction to vaccine injections in order to envisage a new vaccination protocol which prevents the solid tumor formation. To better appreciate this reaction with respect to vaccine injection timing we consider the plots of the relevant quantities separately for each schedule and we mark the vaccination times. As in [16] we consider, for the *S*_1 _statistical samples, the mean values, total number of immune cells and cancer cells in the lattice as function of time. These quantities are plotted in Figures 6, 7, 8, 9, respectively for Very Late, Early, Late and Chronic, for each schedule. Figure 5 show the same quantities for the untreated mice in *S*_1_.

The error analysis shows that in the regions where the sample has strong statistical significance (i.e. there still is a suffcient large number of mice which has not formed a solid tumor), the standard deviation always reaches a maximum of 5–8% for all entities.

First of all we notice that *very late *and *untreated *protocols show similar curves. Thus *very late *schedule is not relevant as it does not affect tumor growth. We will not discuss further this schedule.

*Early *and *late *protocol show a similar general behavior for all quantities. The *chronic *protocol shows a behavior which is different from all the others protocols.

This last three schedules show an initial *transient phase *characterized by a strong cytotoxic response which corresponds to a drastic reduction of cancer cells. This initial, burst like, transient phase appears also, for all the three schedules, in T helpers, cytotoxic T cells, B cells and antibodies. The antibodies grow immediately after the first vaccine injection. This effect is due to the antibodies released by vaccine cells. The general behavior is similar for all the schedules but there are significant differences:

*i) *the vaccine's effect on the growth of cancer cells appears after the second cycle of vaccination;

*ii) *cancer cells drop off roughly two months after the end of the early schedule, while in the late protocol the drop begins in between second and third cycle of vaccination;

*iii) *the same delay can be observed in the other entities behavior (except for natural killer and dendritic cells as explained in [16]);

*iv) *Cytotoxic T cells response is higher in late protocol than in early one. This is probably due to the fact that in late vaccination there are many more cancer cells than in the early;

*v) *Antibody response is more powerful in *early *than *late*. This is crucial for controlling tumor progression [6]: early treated mice survive about 20 weeks more than late treated mice. Early protocol is then successful in delaying the formation of the solid tumor via humoral response.

As already mentioned *chronic *schedule plots show a transient phase as in other schedules. This is followed by a quasi steady phase. All the plots, during the quasi steady phase, are mostly flat and characterized by small humps with maximum at the end of each vaccination cycle. The number of antibodies, after the initial increase, keeps constant. This show, as found in the in vivo data, that cancer growth is controlled by antibodies. The number of cancer cells is always in the range 10^3 ^÷ 10^4^.

### 4.3 Computer aided search for a new schedule

The search for a new schedule can be envisaged from the results, previously shown, of the three protocols giving, at least, an initial positive response, i.e. *early*, *late *and *chronic*.

**Figure 10:**
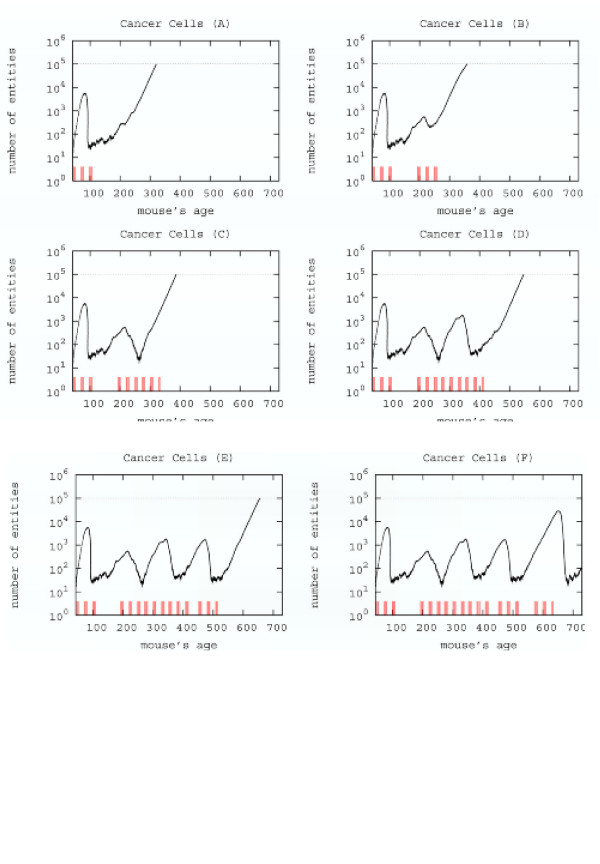
Searching a new schedule: results after early cycles for a single mice. Red ticks above *x *axis represent the timing of vaccine administration.

This search is driven by the following observation: cancer cells drop off roughly two weeks later the last vaccine injection of the third cycle of the early vaccination. Following this observation we randomly choose one mouse and provide a *complete early vaccination, i.e. three cycles *roughly two weeks before the observed minimum of cancer cells. The time of the second vaccination was chosen from the plots of early schedule of the mice, Figure 10(a). Figure 10(b) shows that the repeated early vaccination schedule is not able to stop cancer cells from growing in number. Another complete early vaccination needed to decrease the number of cancer cells as shown in Figure 10(c). To control the tumor growth up to the end of simulation we were again forced to apply twice a complete early vaccinations as shown in Figure 10(d,e,f). The time setting for injections was done heuristically and many trials were necessary. After a number of attempts we envisage a possible alternative therapy. We then tested it in-silico for all mice of sample *S*_1 _and then for all mice of sample *S*_2_.

**Figure 11:**
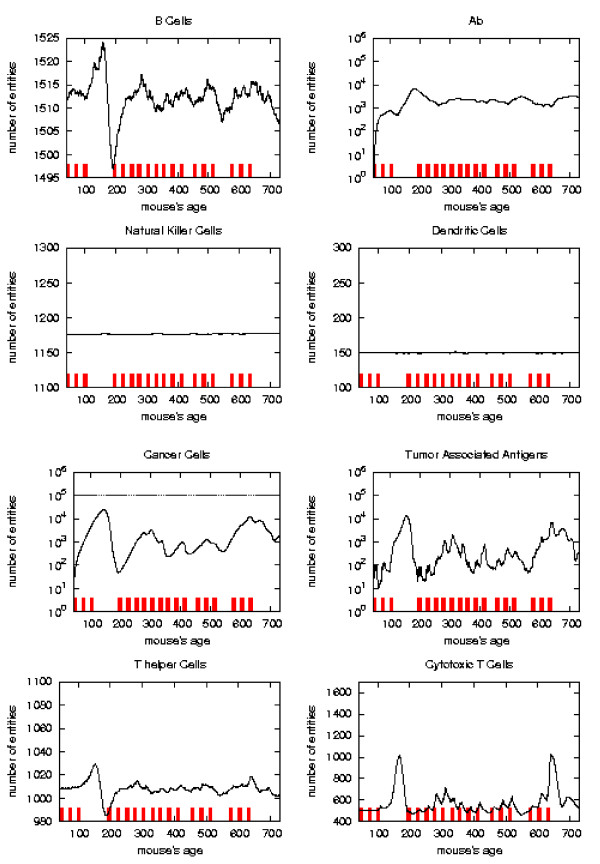
Immune response. The immune response activation due to the vaccine effect is shown for a possible vaccination schedules, versus time in days. Red ticks above *x *axis represent the timing of vaccine administration.

We get the tumor control for 85% of mice of the sample with the schedule shown in Figure 11, which shows the mean values for all mice of sample *S*_1 _in order to compare with previous figures.

This schedule is able to control the tumor formation with a number of vaccine injections 30% less than the *chronic *one.

## 5 Conclusion

We presented an *in silico *search for effective cancer vaccination protocols using a model describing the action of a tumor vaccine in stimulating immune response and the ensuing competition between the immune system and tumor cells. This model applies to the very early stage of tumor genesis, i.e. before a solid tumor is formed. *In silico *experiments show excellent agreement with *in vivo *experiments both for the time of formation of solid tumor in mice and the role of antibody response in controlling tumor growth [6,8]. The model and its computer implementation are very flexible and new biological entities, behavior, and interactions can be easily added. This helps achieving a realistic description of the immune responses that target solid tumor formation.

We found that a possible protocol which prevents solid tumor formation for the mice lifetime and uses a number of vaccine injections 30% less than Chronic schedule. This result shows that, at least in principle, a computerized mathematical model can be used to search for better protocols. The vaccination schedule we found is better than the Chronic one. However the question if this schedule is optimal, i.e. is the effective schedule with the minimum number of injections, or nearly-optimal is still open. The answer to this question is not trivial. First one should properly define the biological meaning of an optimal schedule; then an optimal/nearly optimal solution can be found with different techniques available in the *mathematical market*. We plan to further investigate this point using alternative strategies, or optimal search algorithms like simulated annealing or genetic algorithms, taking into account biological constraints.

Moreover the model we used is still naive and can be improved greatly. We are working to a more detailed model to dissert the contribution of individual vaccine's components to the protective immune response. We plan also to consider a multi-organ model in order to consider metastasis formation, and the vaccine's effect on metastasis. Work in these directions is in progress and results will be published in due course.

### Note

^1^

^2^Paul E. Black, "Hamming distance", from Dictionary of Algorithms and Data Structures, Paul E. Black, ed., NIST. 
